# Automated long-term field monitoring reveals diel and seasonal activity patterns of *Drosophila suzukii*

**DOI:** 10.1371/journal.pone.0354134

**Published:** 2026-08-06

**Authors:** Felix Briem, Jean Fred Fontaine, Ralf Neukampf, Heidrun Vogt

**Affiliations:** 1 Julius Kühn-Institut, Institute for Plant Protection in Fruit Crops and Viticulture, Dossenheim, Germany; 2 Central Institute for Decision Support Systems in Crop Protection (ZEPP), Bad Kreuznach, Germany; 3 Julius Kühn-Institut, Institute for Strategies and Technology Assessment, Kleinmachnow, Germany; University of Carthage, TUNISIA

## Abstract

*Drosophila suzukii* is a globally invasive pest causing substantial losses in soft fruit production systems. While diel activity has been studied under laboratory conditions, long-term high-resolution field data remain scarce. Here, we present a multi-year automated monitoring dataset from four field sites in South-West Germany (2017–2019) using traps sampling at 3-hour intervals, combined with high-resolution environmental data. Across the study period, *D. suzukii* exhibited strong seasonal dynamics, with low activity in winter and early spring, increasing in late spring and summer, and declining in autumn. Diel activity patterns were not constant but varied with seasonal abundance and environmental conditions. During periods of high activity, captures were concentrated in daylight hours and often showed bimodal peaks in the morning and late afternoon. In contrast, during low-abundance periods, diel patterns were weak or absent. Temperature effects on capture rates were season-dependent, showing positive associations in late spring and autumn but negative associations in summer. Rain consistently reduced capture rates across all seasons, whereas wind showed weaker and less consistent effects. Overall, our results show that diel activity of *D. suzukii* emerges under specific seasonal and environmental conditions rather than being a fixed behavioural trait. The custom-built automated trapping system proved reliable for high-frequency field monitoring and provides a scalable approach for other small insect monitoring systems. These findings emphasise the importance of considering both temporal resolution and environmental context when interpreting trap data and designing pest management strategies.

## Introduction

*Drosophila suzukii* Matsumura (Diptera: Drosophilidae), commonly known as Spotted Wing Drosophila, is an invasive pest of major economic importance in soft fruit production systems worldwide [1,2]. Its economic impact is particularly evident in berry and cherry production systems, where significant yield losses have been reported [2–4]. Since its introduction from its native range in Southeast Asia, it has rapidly expanded across multiple continents, driven by its ability to infest ripening fruit prior to harvest, resulting in substantial crop losses in temperate fruit-growing regions, including Europe [1,3,5–7]. In Germany*, D. suzukii* was first detected in 2011 [8,9] and subsequently established in major fruit-growing regions. Widespread establishment occurred following the mild winter of 2013/2014, which facilitated population build-up and long-term persistence [10,11]. Since then, increasing population pressure has required intensified monitoring and management efforts in soft fruit and cherry production systems [6,11,12]. This invasion success is facilitated by ecological plasticity, high dispersal capacity, and tolerance to a wide range of climatic conditions [1,6,13].

Effective management of *D. suzukii* requires a detailed knowledge of both seasonal population dynamics and fine-scale daily activity patterns, particularly because control measures are most effective when timed with periods of pest activity. While seasonal population dynamics are well documented through long-term monitoring studies [11,12,14], diel activity under natural field conditions remains comparatively poorly quantified. Most existing knowledge is derived from laboratory or semi-field experiments under simplified light–dark regimes [15–17], which may not capture the complexity of field environments. Moreover, behavioural responses can differ substantially between laboratory and field conditions [18–20]. Field studies further suggest that daily movement and dispersal patterns can vary dynamically across habitats and time of day, highlighting the importance of measurements under natural field conditions [20,21]. Moreover, temperature is a key driver of both activity and population dynamics in *D. suzukii* [22–25], reinforcing the need to integrate environmental variability into field-based assessments of behavioural patterns. A key limitation of current field studies is their low temporal resolution. Standard trapping typically provides daily or multi-day aggregated counts, limiting the ability to resolve short-term and diel fluctuations [26,27]. Although diel activity has been demonstrated experimentally, its expression under natural field conditions across seasons remains insufficiently resolved under field conditions.

Recent advances in automated monitoring systems have enabled higher-frequency sampling and new opportunities for continuous insect monitoring under natural field conditions [28–30]. These systems integrate low-cost hardware and sensor technologies, including camera- and microcontroller-based approaches [31,32], and increasingly incorporating machine learning methods for automated insect classification [33–35]. Despite these advances, long-term high-frequency datasets resolving diel activity of *D. suzukii* remain scarce, and multi-year validation of automated systems under field conditions is still limited [36]. Consequently, hybrid approaches combining automated sampling with manual validation remain essential for robust ecological inference. However, most automated systems primarily focus on detection and counting, while often not preserving specimens for downstream biological analyses.

To address these gaps, we developed an automated trapping system capable of sampling insects at 3-hour intervals throughout the day. The system was deployed at four relevant host plant sites at the experimental fields of the Julius Kühn-Institut (JKI) in Dossenheim, South-West Germany over a two-year period to quantify seasonal and diel activity patterns of *D. suzukii* under natural field conditions. Trap catches were combined with high-resolution local weather data to assess how abiotic factors and phenological progression shape activity dynamics across space and time.

The aim of this study was to quantify high-resolution seasonal and diel activity patterns of *D. suzukii* under field conditions and to assess how these patterns are modulated by environmental variables and spatial context. We hypothesised that (i) activity exhibits strong seasonal structuring, (ii) diel structuring varies with seasonal abundance, and (iii) environmental variables such as temperature and rainfall are associated with variation in capture rates.

## Methods

### Trap design and automated sampling

**Fig 1:**
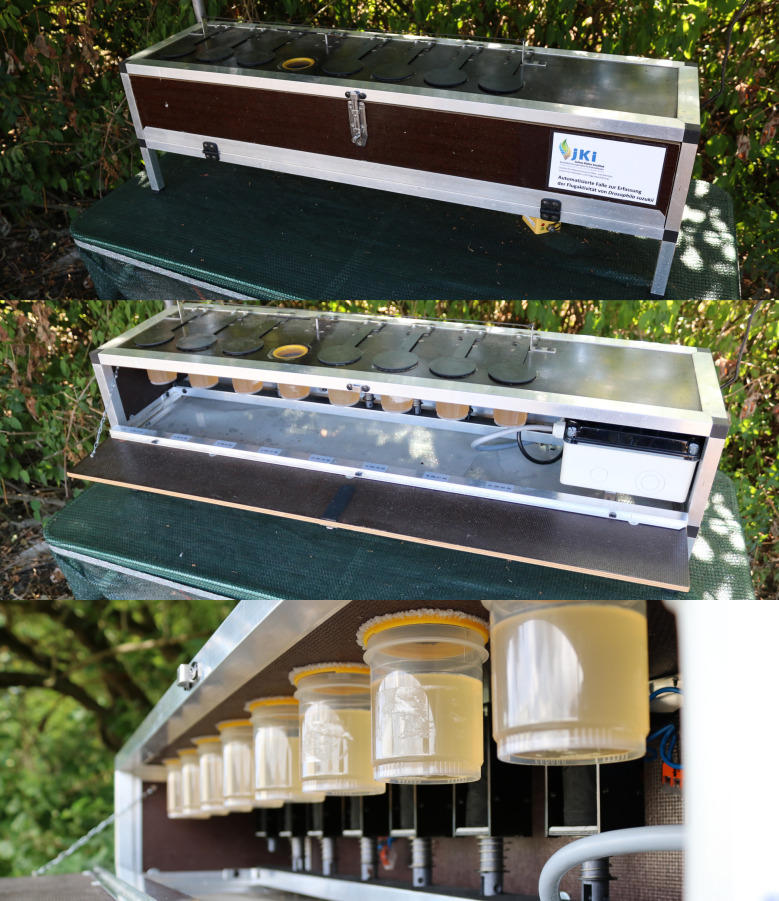
Custom-built automated multi-compartment trap used for high-resolution monitoring of *Drosophila suzukii.* The Arduino-based control system sequentially opens eight baited sampling units, enabling automated insect collection in predefined 3-hour intervals over a 24-hour cycle. Each sampling unit consists of a collection cup filled with an apple cider vinegar–water mixture (40:60 v/v) used as attractant and a lightweight carbon-fibre flap controlling access to the cup. The upper panel shows the trap in its closed field-operating state, the middle panel shows the trap opened during servicing and replacement of collection cups, and the lower panel shows a close-up of a sampling unit and the internal mechanism.

Four identical automated multi-compartment traps were constructed using low-cost electronic components and an Arduino UNO-based control system (see S1 Table for a full list of components). Each trap consisted of eight independently operated sampling units, enabling sequential insect collection in 3-hour intervals over a continuous 24-hour cycle (Fig 1). The trap frame (100 × 25 cm) was built from aluminium profiles and designed for long-term outdoor deployment under field conditions. Each sampling unit contained a 120 ml cup filled with an apple cider vinegar (ACV)–water solution (40:60 v/v) supplemented with a small amount of detergent to reduce surface tension. The ACV–water mixture was used as bait because it is a commonly used attractant for *D. suzukii* monitoring under field conditions [9,37]. The dilution was chosen to balance attractiveness and practicality for long-term automated sampling while reducing excessive non-target catches observed in more complex bait formulations [9,37].

Each opening was covered with a 2 mm mesh gauze and a lightweight carbon-fibre flap to reduce non-target entry while allowing volatile attraction. The flap movement was controlled by 12 V solenoid actuators connected via an eight-channel relay system. Timing was regulated using a DS3231 real-time clock module, enabling automated switching between sampling intervals. All electronic components were housed in a weatherproof enclosure, and traps were powered via a mains-to-DC conversion system. Traps were installed approximately 1 m above ground and protected by a roof to reduce rainfall and overheating effects. Sampling cups were replaced and processed daily on weekdays.

### Study sites and period

**Fig 2:**
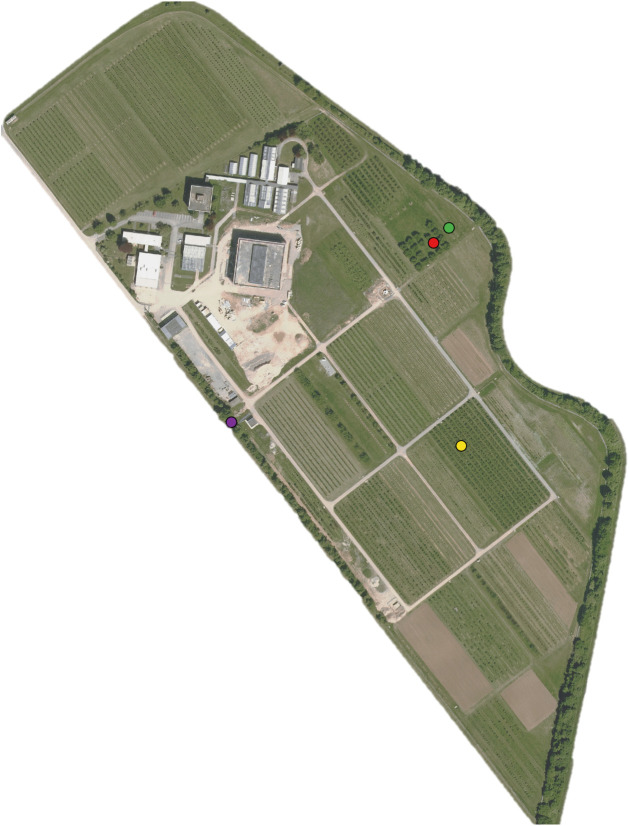
Spatial arrangement of the four automated trapping systems at the experimental field of the Julius Kühn-Institut (JKI) in Dossenheim, Germany. Trap locations include hedgerow vegetation (purple), a raspberry plot (green), an isolated cherry tree (cv. Sam; red), and a cherry plantation (cv. Regina; yellow). Aerial image: © LGL, www.lgl-bw.de, dl-de/by-2-0, https://owsproxy.lgl-bw.de/owsproxy/ows/WMS_LGL-BW_HIST_DOP_2010-2019?.

To investigate seasonal and diel activity of *Drosophila suzukii* under field conditions, four automated traps were deployed over a two-year period from July 2017 to July 2019 at the experimental fields of the JKI (Fig 2). The study sites represented different host plant and vegetation types, including hedgerow vegetation, a small raspberry plot (cv. Himbotop), a semi-dwarf cherry stand (cv. Sam; planted in 1977), and a 0.5 ha cherry orchard (cv. Regina; planted in 2007). Sites differed in vegetation structure and management intensity but were all embedded within the same experimental field complex. No insecticides were applied at any site during the study period. The Regina trap was temporarily covered during fungicide and foliar fertilizer applications to prevent contamination of the trap; all other sites were managed under extensive, low-input conditions.

### Weather data and environmental variables

Microclimatic conditions (temperature and humidity) were recorded using Tinytag Plus 2 data loggers equipped with radiation shields and installed adjacent to each trap. Additional meteorological data (temperature, humidity, rain and wind speed) were obtained from the JKI weather station at 5-minute intervals (Adolf Thies GmbH & Co. KG, Göttingen, Germany).

All environmental variables were aggregated to 3-hour intervals corresponding to trap sampling periods. Temperature and humidity were averaged, while rainfall was summed. Station-based and microclimate measurements were highly correlated (S2 Table) and were therefore combined in the analyses; missing logger data were imputed using corresponding station data. Rainfall values were log-transformed prior to analysis to reduce the influence of extreme values.

### Phenological classification

Phenological periods were defined following Briem et al. (2018) [11] (Table 1). Winter and early spring were excluded from statistical analyses because capture rates were near zero, resulting in strong zero inflation.

**Table 1:** Classification of phenological periods.

Season	Date
Winter	January 1 – March 11
Early Spring	March 12 – May 15
Late Spring	May 16 – July 29
Summer	July 30 – October 7
Autumn	October 8 – December 31

### Statistical analysis

Capture data were analysed using generalized linear mixed models (GLMMs) with a negative binomial error distribution and a log link function to account for overdispersion [38,39]. Device identity, phenological period (late spring, summer, autumn), and time of day (eight 3-hour intervals) were included as random effects. Environmental variables and sex were included as fixed effects. To evaluate the contribution of individual model components, reduced models were compared to the full model using Akaike Information Criterion (AIC), with lower values indicating improved model fit. Model structures and corresponding AIC values are provided in S3 Table. All analyses were conducted in R version 4.3.1 [40], and figures were generated using ggplot2 v3.4.2 [41].

## Results

### Seasonal and spatial dynamics of *Drosophila suzukii* abundance

**Fig 3:**
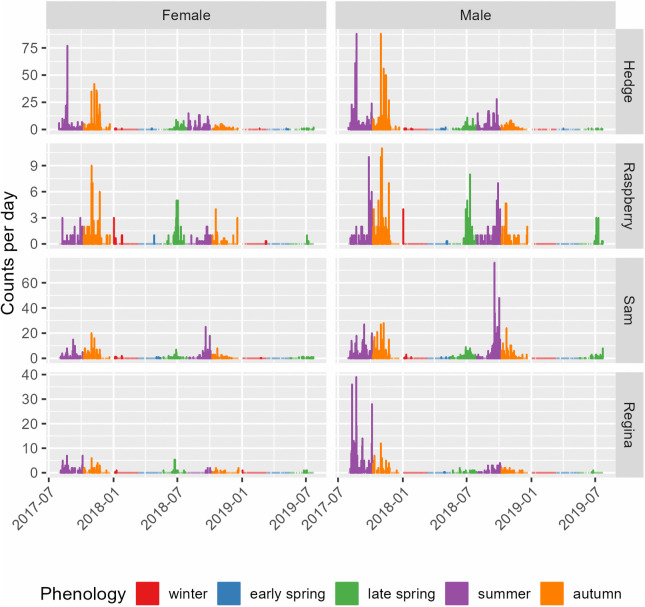
Seasonal dynamics of *Drosophila suzukii* captures at four field sites. Daily numbers of male and female adults recorded by automated traps from 2017 to 2019. Trap locations include hedgerow vegetation, raspberry, cherry cv. Sam, and cherry cv. Regina.

A total of 9,090 *D. suzukii* individuals were captured during the monitoring period from July 2017 to July 2019, comprising 2,974 females and 6,116 males (Table 2). Capture rates showed strong temporal variation across seasons and sites (Fig 3). Across all sites, captures were consistently low during winter and early spring in both years, followed by a marked increase in late spring and sustained high abundance throughout summer and autumn. This seasonal pattern was consistent across sites and years, indicating a recurrent phenological cycle (Fig 3).

**Table 2:** Annual sum of *Drosophila suzukii* catches (female vs. male).

Year	Female	Male	Total	Male/Female-ratio
7/2017 − 6/2018	2,038	3,882	5,920	1.90
7/2018 − 6/2019	936	2,234	3,170	2.39

Substantial spatial differences were observed among trapping locations. The Hedge site consistently recorded the highest capture rates across most of the study period, with pronounced peaks during summer and autumn. The Sam site also showed high abundance, particularly during summer 2018. In contrast, Raspberry consistently exhibited the lowest capture rates, while Regina showed intermediate levels but generally remained below Sam, especially during 2018. These spatial differences were consistent across seasons and sexes (Fig 3).

A decline in total abundance was observed between the two monitoring years, with captures decreasing from 5,920 individuals (July 2017–June 2018) to 3,170 individuals (July 2018–June 2019) (Table 2). This reduction was consistent across sites and sexes. Males consistently outnumbered females, accounting for approximately two-thirds of total captures (male-to-female ratio ≈ 2.06:1). Despite this difference in magnitude, seasonal patterns of males and females were highly synchronized across sites and years (Fig 3).

### Diel activity patterns

**Fig 4:**
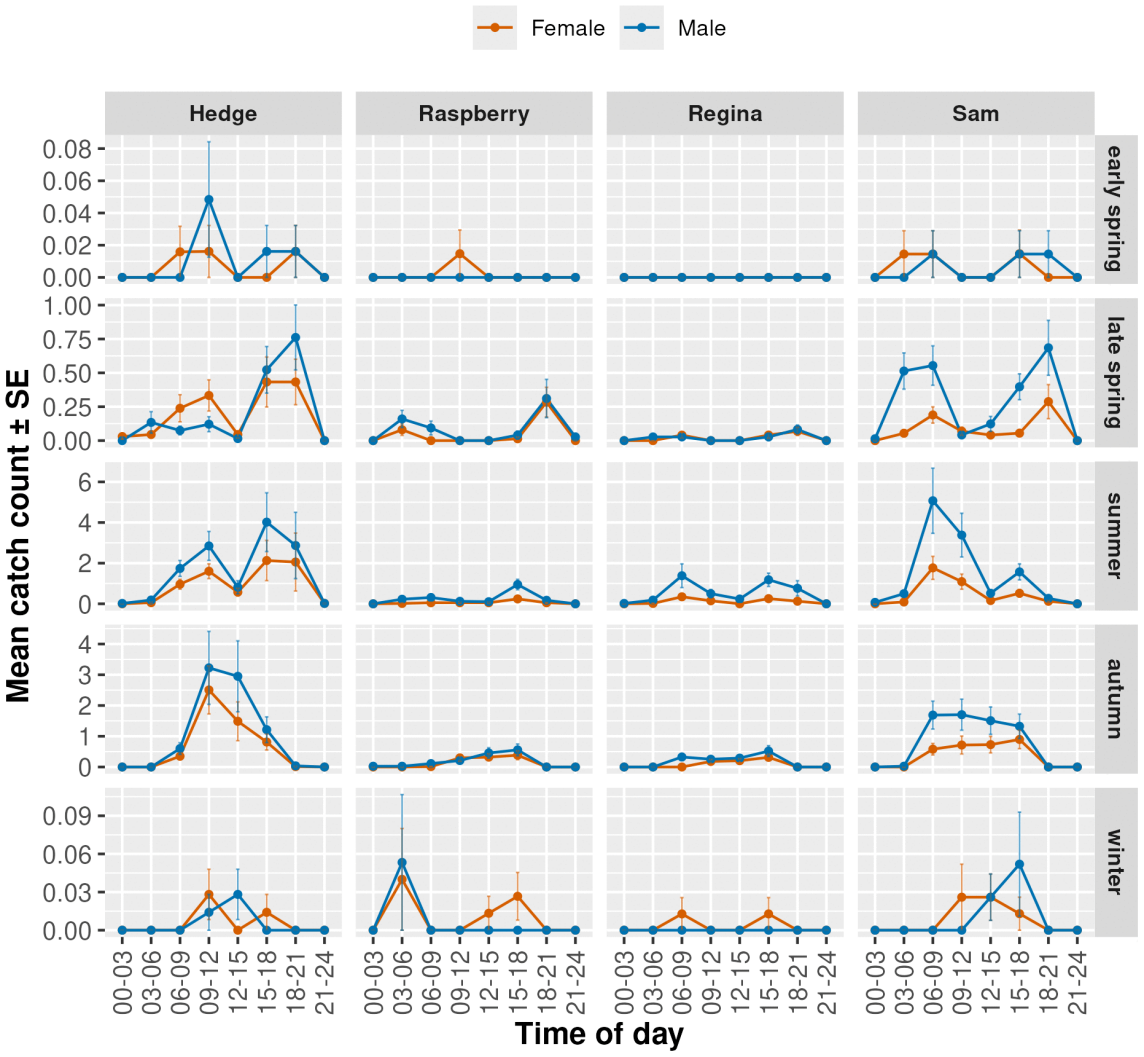
Mean (± SE) *Drosophila suzukii* captures across phenological periods, trap devices, sex, and time of day. Values represent mean captures per sampling interval. Facets separate phenological periods (rows) and trap devices (columns), highlighting seasonal and spatial variation in diel activity patterns. Error bars indicate standard errors.

Diel activity of *D. suzukii* showed clear and repeatable temporal structuring during periods of elevated seasonal abundance (Fig 4). Activity was generally concentrated during daylight hours, whereas night-time captures remained low. Across seasons, diel activity followed a systematic progression. During winter and early spring, captures were rare and diel patterns were weak or absent. In late spring, activity increased and became distributed across most daytime hours. The strongest and most structured diel patterns occurred in summer, with pronounced daytime activity and a characteristic midday decline. In autumn, activity declined slightly and became more evenly distributed across daytime hours (09:00–18:00 h) (Fig 4). At Hedge and Sam, late spring and summer showed elevated activity in both morning (06:00–12:00 h) and late afternoon (18:00–21:00 h), suggesting a bimodal structure. Raspberry and Regina showed similar temporal patterns but at substantially lower abundance. In summer, rapid increases in activity occurred after sunrise (06:00–09:00 h). At Sam, males reached the highest capture rates of the entire study period, with peak activity between 09:00 and 12:00 h. Hedge showed a similar pattern, including a midday decline. Raspberry and Regina remained low but retained detectable diel structure. In autumn, diel patterns remained evident but were less clearly bimodal, with activity distributed across daytime intervals. Winter and early spring showed no consistent diel structure due to very low capture rates. Across all phenological periods, males and females showed highly similar temporal patterns, indicating that sex-related differences were primarily quantitative rather than temporal (Fig 4).

### Environmental and sex-specific effect

**Fig 5:**
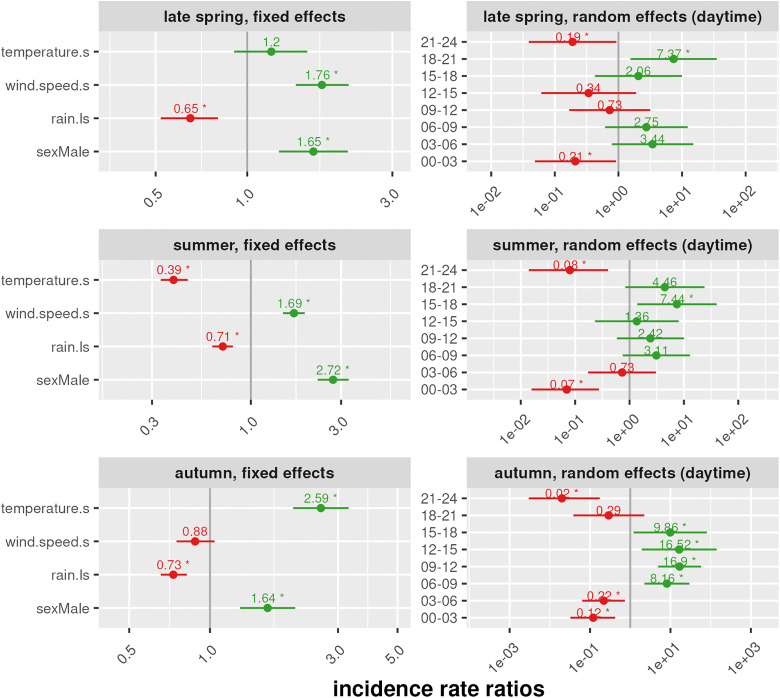
Phenology-specific effects of environmental variables and sex on *Drosophila suzukii* capture rates. Incidence rate ratios (IRR) from negative binomial generalized linear mixed models are shown for temperature, wind speed, rainfall, and sex across late spring, summer, and autumn. IRR values indicate multiplicative effects on expected capture rates (IRR > 1 = increase; IRR < 1 = decrease). Error bars indicate 95% confidence intervals; effects are considered supported when confidence intervals do not overlap 1. Asterisks indicate statistical significance at α = 0.05.

Environmental variables showed clear seasonal variation in their associations with capture rates (Fig 5). Temperature effects were positive in late spring (IRR = 1.48, p < 0.05) and autumn (IRR = 2.36, p < 0.05), but negative in summer (IRR = 0.38, p < 0.05), indicating a seasonal reversal in temperature–activity relationships. Rainfall consistently reduced capture rates across all phenological periods (late spring: IRR = 0.73; summer: IRR = 0.62; autumn: IRR = 0.77; all p < 0.05). Wind effects were weaker and inconsistent, showing positive associations in late spring and summer but negative effects in autumn. Males were significantly more frequently captured than females across all seasons, with the strongest effect in summer (IRR = 2.87), followed by late spring (IRR = 1.78) and autumn (IRR = 1.74). Despite differences in magnitude, both sexes exhibited comparable seasonal and diel patterns (Figs 3–4). Daytime activity patterns mirrored diel results: late spring showed morning and evening peaks, summer showed strong daytime activity with a midday decline, and autumn showed broad daytime distribution.

### Temporal variation across scales

Seasonal, diel, and spatial variation in *D. suzukii* activity occurred simultaneously across the study period. Higher seasonal activity coincided with more pronounced diel structuring, whereas periods of low activity showed weak or absent diel patterns. The strength of temporal structuring varied among sites, with the most pronounced diel patterns observed at Hedge and Sam.

## Discussion

Monitoring insect pests in agricultural systems is essential for effective pest management. Automated monitoring approaches are developing rapidly but still face limitations in scalability and long-term field deployment [28]. Although computer vision and deep learning methods show strong potential, most systems remain restricted to controlled or short-term applications [28]. As a result, high-resolution long-term datasets of insect activity under natural field conditions remain scarce, particularly for *Drosophila suzukii* [29,42]. Here, we present a high-frequency, multi-year dataset based on automated trapping across four sites, enabling the simultaneous assessment of seasonal, diel, environmental, and spatial variation in field populations.

Across all study sites, *D. suzukii* showed strong seasonal dynamics, with consistently low activity during winter and early spring and increasing abundance from late spring into summer. These seasonal patterns were consistent across years and locations and reflect the strong dependence of *D. suzukii* population dynamics on climatic conditions. Similar seasonal dynamics under temperate Central European conditions have been reported in long-term monitoring studies from Germany (e.g., 11). Overwintering survival represents a key bottleneck determining spring population size. During the reproductive period, moderate temperatures (approximately 20–25 °C) and high humidity promote population development, whereas heat stress and dry conditions reduce survival and reproduction [22,43,44]. Consequently, strong interannual variation is commonly observed in invasive populations [1,6,13,45].

Total captures declined between study years (5,920–3,170 individuals), with consistent reductions across all sites and sexes, suggesting a system-wide population decrease rather than local effects or sampling artefacts. Spatial differences in abundance were persistent throughout the study. The Hedge and Sam sites consistently showed higher capture rates than Raspberry and Regina, indicating more favourable conditions for *D. suzukii*. This pattern aligns with previous findings that structurally complex habitats such as hedges and forest edges support higher activity due to more stable microclimates and resource availability [6,11]. In contrast, more open and sun-exposed sites such as Raspberry and Regina likely provide less suitable microclimatic conditions. Additionally, differences in host phenology may contribute to spatial variation, potentially creating temporal mismatches between peak fruit availability and fly activity [46,47]. Despite this, the consistency of spatial patterns across years suggests robust site-level effects.

Diel activity of *D. suzukii* showed clear and repeatable temporal structuring during periods of elevated seasonal abundance [15,17,20]. Activity was generally concentrated during daylight hours, whereas night-time captures remained low. Across seasons, diel activity showed a systematic progression: during winter and early spring, captures were rare and diel patterns were weak or absent; in late spring, activity increased and became distributed across most daytime hours; the strongest and most structured diel patterns occurred in summer, with pronounced daytime activity and a midday decline; in autumn, activity declined slightly and became more evenly distributed across daytime hours. At Hedge and Sam, late spring and summer showed elevated activity in morning (06:00–12:00 h) and late afternoon (18:00–21:00 h), suggesting bimodal structure. In summer, rapid increases in activity occurred after sunrise (06:00–09:00 h), with peak activity at Sam between 09:00 and 12:00 h. Hedge showed similar patterns including a midday decline. Raspberry and Regina showed comparable temporal patterns but at lower overall abundance. In autumn, diel structuring remained evident but less clearly bimodal, with activity broadly distributed across daytime intervals. Winter and early spring showed no consistent diel structure due to very low capture rates. This seasonal variation in diel structure likely reflects a combination of changes in population activity levels, environmental conditions, and sampling sensitivity. When overall capture numbers are low, diel patterns are inherently more difficult to resolve, whereas higher seasonal activity allows clearer detection of temporal structure. Trap-based data therefore reflect relative activity patterns rather than absolute abundance, as catches integrate insect activity, environmental conditions, and attractant-mediated responses [27,48].

Environmental drivers showed clear but seasonally varying effects. Temperature effects were positive in late spring and autumn but negative in summer. Rain consistently reduced captures across all seasons, while wind showed weaker and less consistent effects. These patterns highlight strong context dependency in environmental regulation of activity, reflecting physiological and behavioural plasticity in field conditions [22,24,44].

Males were more frequently captured than females across the study period. This difference in magnitude was consistent across seasons but did not translate into differences in temporal patterns. Both sexes exhibited highly similar seasonal and diel dynamics, indicating that sex primarily affects capture probability rather than temporal activity structure. This suggests that observed sex-specific differences are likely related to behavioural differences affecting trap attraction or encounter probability rather than differences in underlying temporal activity rhythms [1,26,48], potentially linked to sex-specific responses to fermentation cues and movement or resource-seeking behaviour [22,23,46].

The high temporal resolution of the dataset enabled detection of fine-scale diel dynamics that would not be possible with lower-frequency sampling approaches. Variation in diel structuring across seasons further highlights the importance of sub-daily sampling for understanding behavioural dynamics in field populations [29,33]. In addition, the automated trapping system preserves physical specimens, enabling downstream morphological and molecular analyses as well as trait-based assessments, thereby linking continuous ecological monitoring with specimen-based approaches.

Previous automated monitoring systems have demonstrated the feasibility of high-frequency insect detection in field environments [32,33,35,42], but often rely on short-term or intermittent sampling. In contrast, this study provides continuous, multi-year, sub-daily resolution data, enabling integrated analysis of seasonal, diel, and spatial processes within a single framework.

Overall, *D. suzukii* activity and diel organisation in agricultural landscapes are structured across interacting temporal and spatial scales. These findings demonstrate that temporal behavioural structure is dynamic and shaped by ecological context. These results have implications for the interpretation of trap data and for pest management strategies. The identification of consistent diel patterns suggests that monitoring and control efforts could potentially be optimised by considering time-of-day variation in activity. The automated trapping system was robust and reliable during extended field deployment and may be adaptable for other insect taxa through the use of alternative attractants or pheromone-based lures.

## Supporting information

S1 Table List of components used per trap. (XLSX)

S2 Table Overall correlation coefficients between the JKI weather station and TinyTag data loggers for temperature and humidity at the four sites. (XLSX)

S3 Table Seasonal captures by season and sex. Model comparison of generalized linear mixed models differing in random-effect structure. Values indicate inclusion (1) or exclusion (0) of each random effect. Device identity, phenology, and time of day were sequentially removed from the full model (base). Model fit was assessed using Akaike Information Criterion (AIC), with lower values indicating better fit.
(XLSX)

S1 Fig Device-specific random effects from phenology-specific GLMMs (late spring, summer, autumn). Points show incidence rate ratios (IRR) with 95% confidence intervals for each trap device (Hedge, Raspberry, Regina, Sam). Estimates represent deviations from the overall intercept and highlight consistent spatial differences in capture rates across phenological periods.
(TIF)
